# The potential of *Antheraea pernyi* silk for spinal cord repair

**DOI:** 10.1038/s41598-017-14280-5

**Published:** 2017-10-23

**Authors:** A. Varone, D. Knight, S. Lesage, F. Vollrath, A. M. Rajnicek, W. Huang

**Affiliations:** 1 Institute of Medical Sciences, University of Aberdeen, Foresterhill, Aberdeen, AB25 2ZD UK; 2 Oxford Biomaterials Ltd., Unit 15, Calaxy House, Newbury, RG19 6HR UK; 3 Department of Zoology, University of Oxford, South Parks Rd, Oxford, OX1 3PS UK

## Abstract

One of the most challenging applications for tissue regeneration is spinal cord damage. There is no cure for this, partly because cavities and scar tissue formed after injury present formidable barriers that must be crossed by axons to restore function. Natural silks are considered increasingly for medical applications because they are biocompatible, biodegradable and in selected cases promote tissue growth. Filaments from wild *Antheraea pernyi* silkworms can support axon regeneration in peripheral nerve injury. Here we presented evidence that degummed *A. pernyi* filaments (DAPF) support excellent outgrowth of CNS neurons *in vitro* by cell attachment to the high density of arginine-glycine-aspartic acid tripeptide present in DAPF. Importantly, DAPF showed stiffness properties that are well suited to spinal cord repair by supporting cell growth mechano-biology. Furthermore, we demonstrated that DAPF induced no activation of microglia, the CNS resident immune cells, either *in vitro* when exposed to DAPF or *in vivo* when DAPF were implanted in the cord. *In vitro* DAPF degraded gradually with a corresponding decrease in tensile properties. We conclude that *A. pernyi* silk meets the major biochemical and biomaterial criteria for spinal repair, and may have potential as a key component in combinatorial strategies for spinal repair.

## Introduction

WHO states that each year about 250,00 to 500,000 people worldwide suffer spinal cord injury, which has no effective treatment^1^. A complex injury response including a fluid-filled cavity surrounded by a glial scar prevents axon regeneration and spontaneous recovery. The consensus in the field is that there is no silver bullet for spinal cord repair; a regeneration strategy should adopt a combinatorial approach, such as a biomaterial scaffold to bridge the cavity combined with growth-promoting factors to encourage neuronal regeneration and electrical stimulation to guide regenerating axons out of the biomaterial scaffold^2,3^. There is strong evidence that autografts derived from the peripheral nervous system support regeneration of spinal cord neurons^4^ but peripheral nerve grafts cause secondary damage at donor nerve sites and not all CNS neurons show a strong regenerative response to such grafts^5^. An attractive alternative strategy to autografts could be the exploration and development of appropriate natural or synthetic bio- and neuro-compatible materials.

Extensive preclinical studies on biomaterials designed for spinal cord regeneration mostly used collagen, fibrin, polyglycolic acid or poly-DL-caprolactone^6^. Silks are a diverse family of natural materials that can be made into structures such as nets, sponges and membranes. Their exceptional structural and mechanical properties as well as an appropriate rate of resorption have led to wide interest for their use in tissue engineering applications^7^. Commercially produced *Bombyx mori* (*B. mori*) silk is widely employed in surgical sutures and has been shown to support modest growth of dorsal root ganglion (DRG) neurons both *in vitro* and *in vivo* ^8,9^. In addition, silk filaments from the wild silkworm *Antheraea pernyi* (*A. pernyi*) have been shown to promote DRG neurite outgrowth *in vitro* and support excellent peripheral nerve regeneration *in vivo* ^10^, and these filaments are known to contain 11 evenly spaced RGD tripeptide repeats per heavy chain fibroin molecule^11^. Many cell types, including neurons, have integrin receptors that can bind to RGD facilitating cell adhesion. Therefore, the RGD tripeptide may encourage cell binding in *A. pernyi* silk filaments from which the sericin coating has been completely removed by degumming^10^.

In our previous work degummed *A. pernyi* filaments (DAPF) known as Spidrex^®^ silk filaments promoted significant axonal regeneration and functional recovery in a rat model of sciatic nerve injury^10^. This prompted us to investigate here whether DAPF have all the relevant properties considered to be key criteria in biomaterial design for spinal cord repair^12,13^. These include: (1) a surface chemistry and topography to facilitate cell adhesion and provide guidance to axonal extension; (2) a minimal host immune response; (3) a stiffness approximating that of spinal cord to minimise glial cell activation and the consequent inflammatory response, combined with sufficient structural stability to avoid rupture during insertion and healing; and 4) a gradual resorption as axons regenerate. Here we report that DAPF appear to fulfil all these criteria suggesting that DAPF as part of a combinatorial strategy may promote axon regeneration and functional recovery after spinal cord injury.

## Results

### *A. pernyi* silk promotes excellent CNS neuron growth

**Figure 1:**
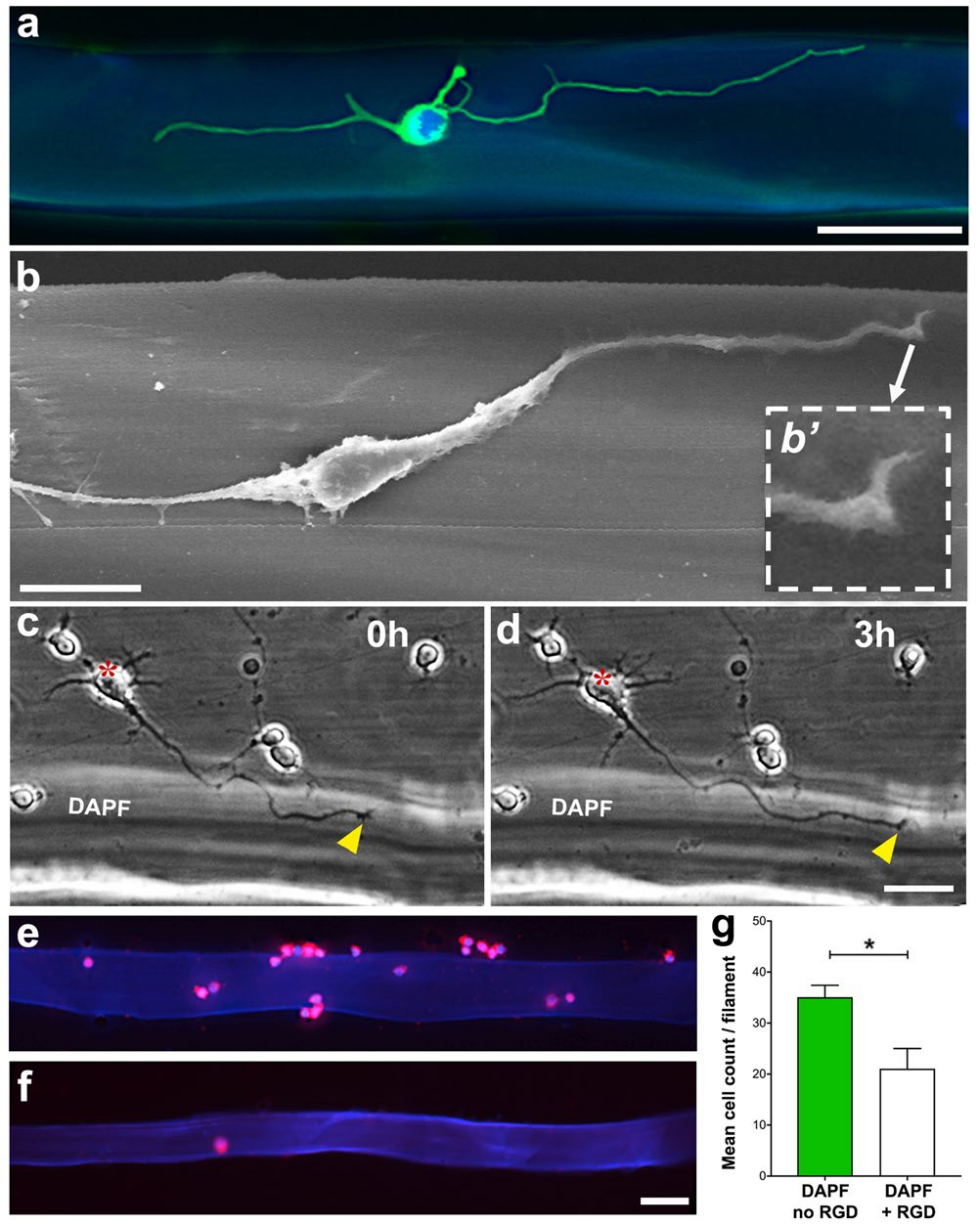
Contact support of DAPF for postnatal rat cortical neuron growth. (**a**) Z-stack fluorescence image of a β-tubulin III labelled cortical neuron after 2 days in culture on DAPF. (**b**) SEM image of a cortical neuron after 5 days in culture on DAPF. (*b’*) A close-up view of an actively growing neuron’s growth cone. (**a** and **b**) did not show the classical growth cone morphology such as that in (**c**), and this is likely due to the fixation process or changes that occurred as the growth cone advanced (see in **d**). (**c** and **d**) Time-lapse sequences, at 0 h and at 3 h, of a growth cone (yellow arrowhead) of a cortical neuron (red asterisk) extending a neurite along the DAPF. (**e** and **f**) Representative fluorescence images of β-tubulin III labelled cortical neurons showing high attachment to DAPF in the absence of RGD peptide (**e**) and low attachment in the presence of RGD peptide (**f**) in the medium. (**g**) Quantitative analysis shows increased attachment of cortical neurons to DAPF, only under conditions where integrins were not blocked by RGD peptide in the medium. *p < 0.05; N = 4 biological replicates (BRs), n = 3 technical replicates (TRs) per BR. Scale bars = 50 µm (**a,c,d,e,f**); 10 µm (**b**).

**Figure 2:**
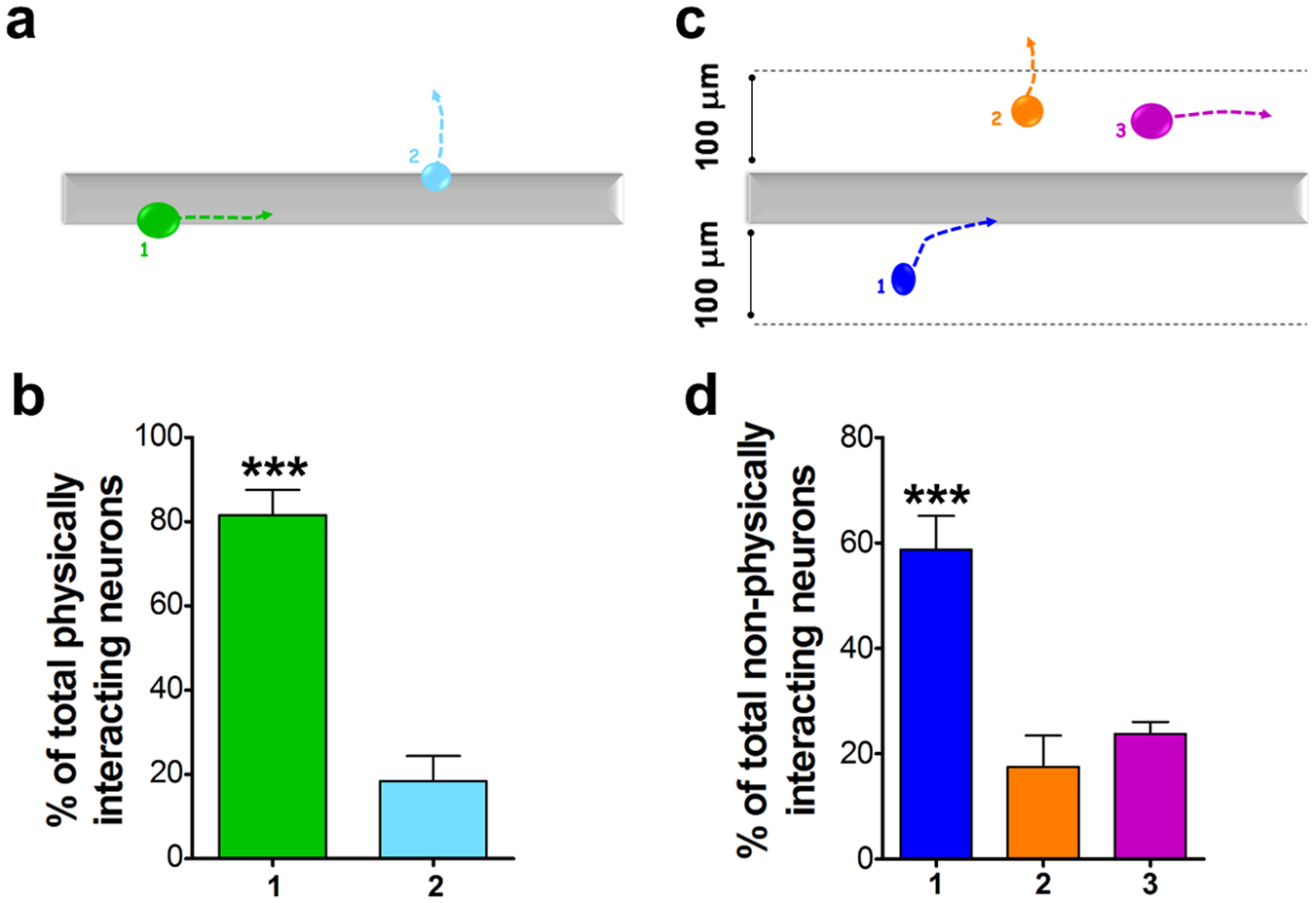
Longest neurite growth directions of cortical neurons in relationship to DAPF. Among cortical neurons physically interacting with DAPF, the percentage of cells with their longest neurites growing on and along DAPF was significantly higher than that of cells with their longest neurites growing away from DAPF (type 1 *vs* type 2 in **a** and **b**; N = 7 BRs, n = 5 TRs; ***p < 0.001, *t-test*). Among cortical neurons not physically interacting with DAPF, we observed a significantly higher percentage of cells growing their longest neurites towards DAPF than those of cells either growing their longest neurites away from DAPF or in parallel with DAPF (type 1 *v*s types 2 & 3 in **c** and **d**; N = 7 BRs, n = 5 TRs; ***p < 0.001). The dotted lines with arrowheads represent the longest neurites of cortical neurons.

To assess whether DAPF promoted cell adhesion and guided neurite growth (contact guidance), we studied the interactions of primary neuron cultures with DAPF using CNS neurons derived from postnatal rat cortex (Figs 1 and 2 and Supplementary Fig. S3) and from embryonic *Xenopus* spinal cord neurons (Supplementary Fig. S3). Confocal and scanning electron microscopies (SEM) showed that cortical neurons extended long neurites along DAPF (Fig. 1a,b) with their growth cones (growing neurite tips) attached to the filaments (Fig. 1b insert). Time-lapse phase contrast microscopy showed that cortical neurons seeded next to DAPF extended neurites towards and onto the filaments. The growth cones of their longest neurites extended surprisingly rapidly, approximately 40 µm in 3 h, along and in close contact with the filaments (Fig. 1c,d). Analysis of the interactions between DAPF and CNS neurons located within a distance of 100 µm of the silk filaments showed that the percentage of neurons with their cell bodies attached to DAPF, i.e. physically interacting with the filaments, was significantly higher than that of neurons with their cell bodies attached to the coverslips, i.e. not physically interacting with the filaments (Supplementary Fig. S3a,b).

For cortical neurons interacting physically with DAPF (Fig. 2a,b) the majority of cells (∼80%) grew their longest neurites on and along the fibres and only ∼20% extended their longest neurites away from DAPF. For those cortical neurons not physically interacting with DAPF (Fig. 2c,d) the majority (∼60%) extended their longest neurites towards DAPF, significantly more than those growing their longest neurites away from (∼17%) or in parallel with (∼23%) DAPF. The mean longest neurite length per cortical neuron growing on DAPF over a 48 h culture period was similar to that of cortical neurons cultured on poly-d-lysine substrates (p > 0.05; Supplementary Fig. S4), a surface used routinely for cortical neuronal cultures.

### RGD tripeptide in *A. pernyi* silk is key for promoting CNS neuron growth

**Figure 3:**
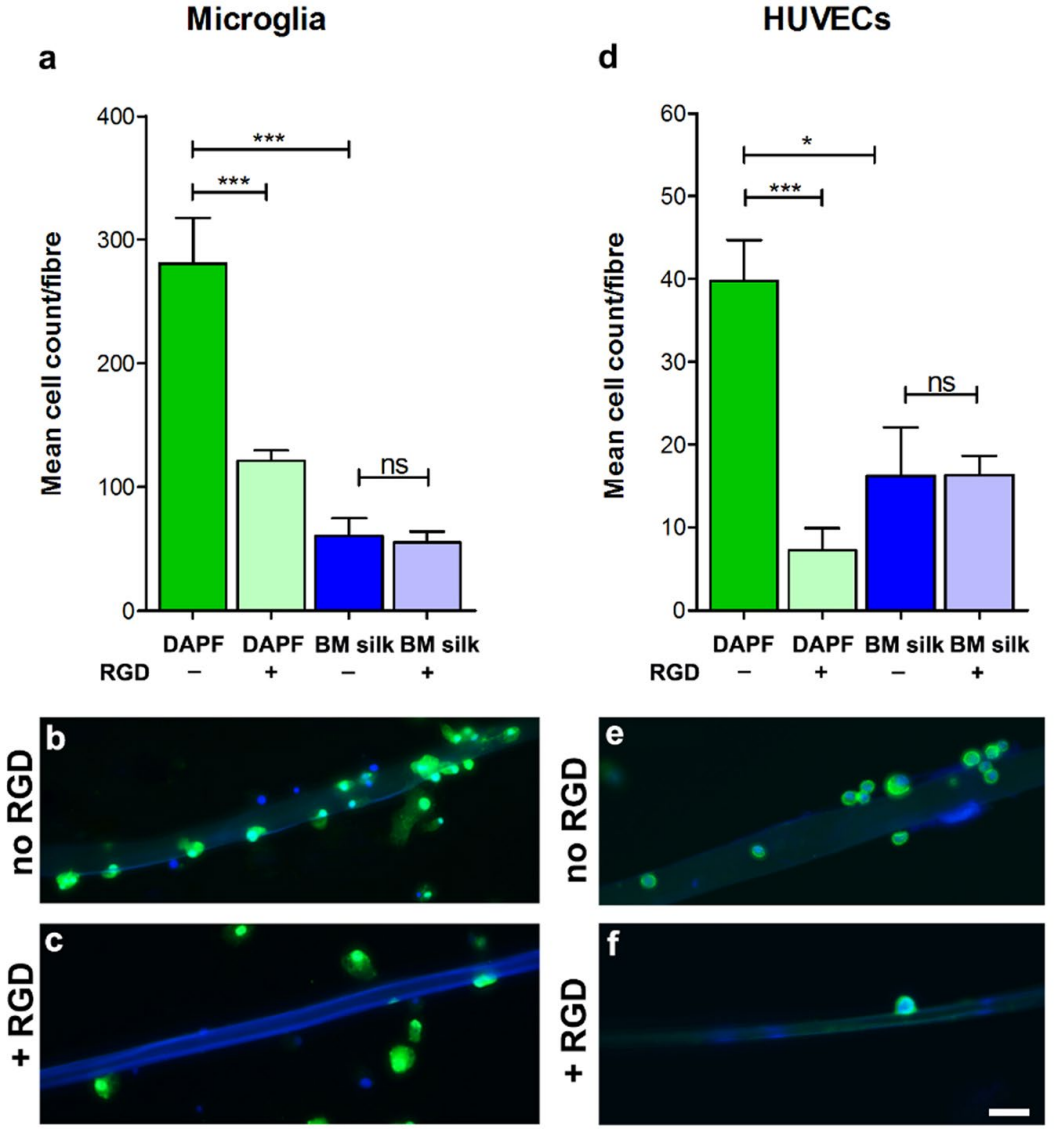
RGD-mediated non-neuronal cell adhesion to DAPF. Mean cell count per filament of (**a**–**c**) microglia and (**d**,**e**) HUVECs on DAPF or BM silk filaments at 1 h after plating, with and without RGD tripeptide added to the culture media. The adhesion of both cell types to DAPF was significantly higher when no RGD was added to culture media compared to that when RGD was added (**a**–**c**). The mean cell count per BM silk filament remained unchanged either with or without RGD added to the culture media (**d**,**e**). In addition, the number of cells adhered to DAPF by both cell types was significantly higher than that with BM silk filaments. N = 3 BRs, n = 3 TRs; *p < 0.05 and ***p < 0.001. Scale bar = 50 µm.

To show that the RGD sequences on DAPF were responsible for the observed preferential adhesion, rat cortical neurons were cultured on either DAPF or *B. mori* filaments for 1 h before fixation to assess their ability to adhere to these filaments. The mean total number of cortical neurons attached to each DAPF was higher than the total for *B. mori* filaments (p < 0.001; Supplementary Fig. S5). In a separate experiment, rat cortical neurons were cultured on DAPF for 1 h before fixation, with or without the addition of RGD tripeptide to act as a competitive inhibitor of integrin mediated cell adhesion. The addition of RGD peptides significantly reduced cortical neuron binding (p < 0.05; Fig. 1e–g). Non-neuronal cell types, such as microglia and blood vessel endothelial cell line HUVECs gave similar results (Fig. 3).

### *A. pernyi* silk induces minimal immune response

**Figure 4:**
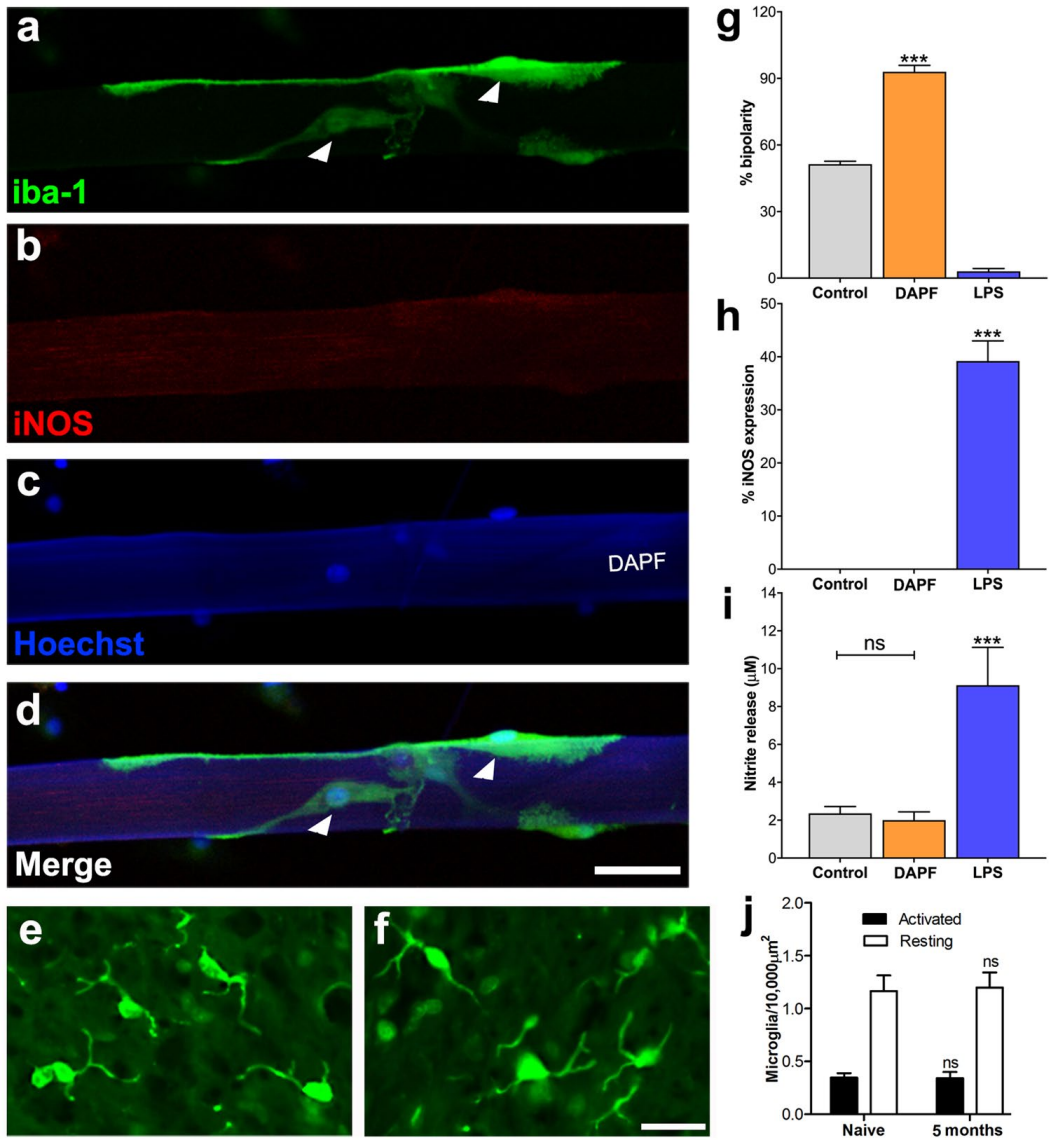
A lack of host immune response of microglia to DAPF. (**a**–**d**) Confocal images of Iba-1 labelled microglia (white arrowhead) interacting with DAPF and showing quiescent morphology (bipolar) with no expression of the pro-inflammatory cytokine iNOS *in vitro*. (**e** and **f**) Representative fluorescence images of microglia in the spinal cord dorsal horn in which there was no implantation (**e**) or where DAPF was implanted for 5 months (**f**). Resting microglia morphology, or with process length double the soma diameter, is unaltered across the two conditions. (**g**) Percentage of total microglia with bipolar (non-activated) morphology was increased significantly on cells cultured on DAPF compared to control (no biomaterial) and LPS (positive controls). Percentages of (**h**) iNOS expression and (**i**) nitrite release are reduced significantly for DAPF compared to LPS and not significantly different from control. (**j**) Counts of the number of activated and resting microglia confirms that there were no morphological changes across all three conditions (N = 3 BRs). For g, h, I: ***p < 0.001; N = 9 BRs, n = 3 TRs/BR. Scale bars = 50 µm.

Our previous work showed that DAPF when used to repair PNS injury in adult rats^10^ induced only a minimal immune response by macrophages. In the CNS, microglial cells are responsible for the immune reaction. Accordingly, we investigated the response of these cells cultured with DAPF. In normal culture (control) conditions, microglia were in a resting, non-activated state indicated by a high proportion showing the bipolar morphology with long processes (Fig. 4g) and the lack of elevated levels of proinflammatory mediators (Fig. 4h,i). However, when exposed to activating agents such as lipopolysaccharide, microglia responded by changing their morphologies into an amoeboid shape with short or no processes (Fig. 4g), and released pro-inflammatory mediators such as iNOS and NO (Fig. 4h), and increased nitrite levels in the culture media (Fig. 4i). Fluorescence microscopy revealed that the percentage of microglia on DAPF showing the bipolar morphology of non-activated cells was significantly higher compared to Control (90% vs 50% p < 0.001; Fig. 4a–d,g). Quantitative image analysis of immunostained microglia exposed to DAPF showed similarly low expression of iNOS, compared to Control (p > 0.05; Fig. 4a–d,h). Using the Griess assay to measure the levels of nitrite in the culture medium we found that microglia cultured in the presence of DAPF released similarly low levels of nitrite compared to Control (p > 0.05; Fig. 4i). In the Control condition described here microglia were cultured without DAPF and without LPS. We also assessed spinal cord microglial responses of uninjured adult rats that had received an implant of DAPF 5 months earlier, counting Iba1 immuno-reactive microglia in their resting or activated morphologies. We found that microglia from both the naïve spinal cords and those that received DAPF implants had similar high proportions of “resting” morphology (p > 0.05; Fig. 4e,j).

### *A. pernyi* silk has suitable mechanical properties for spinal cord repair

**Figure 5:**
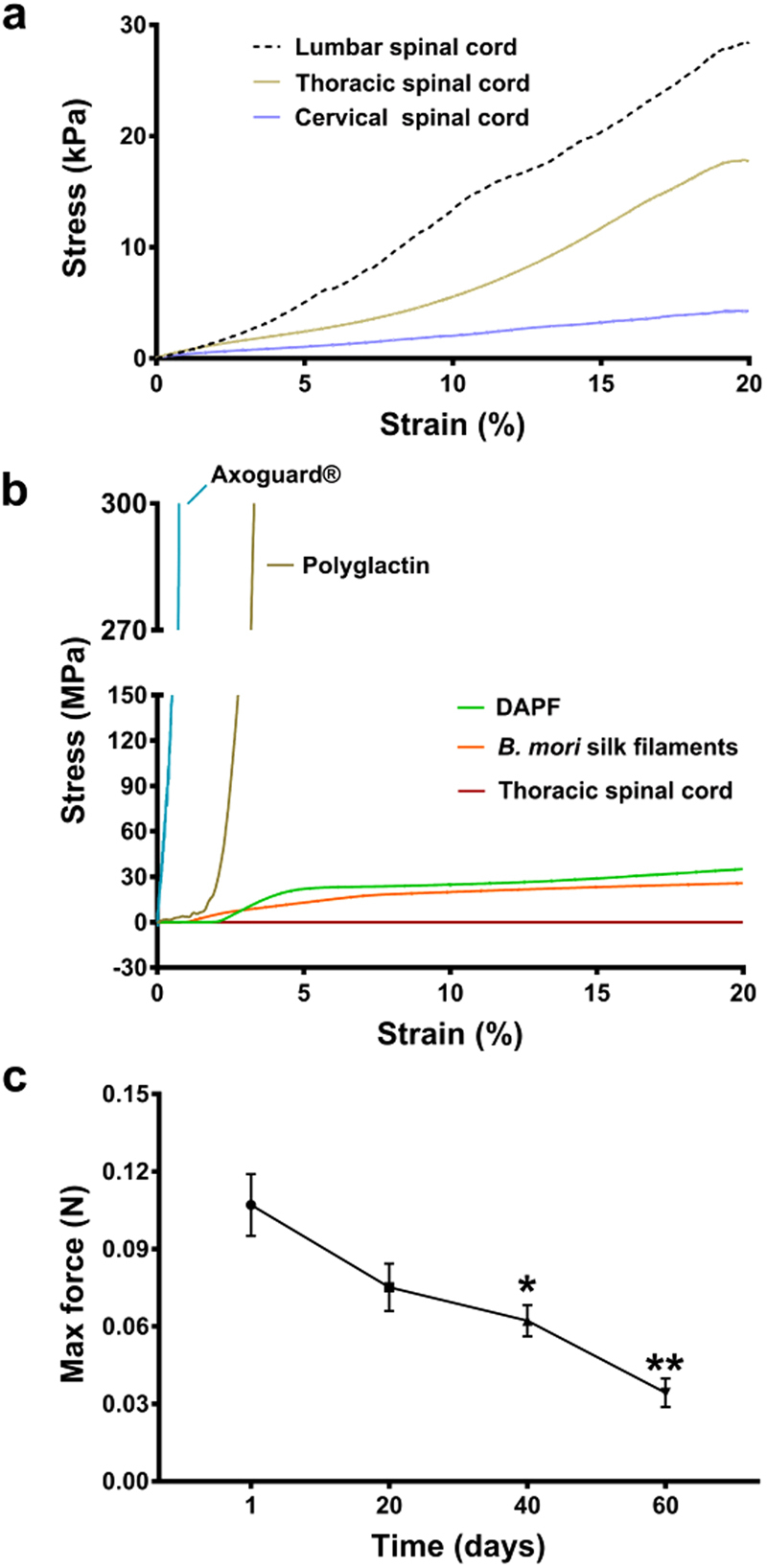
Mechanical properties of DAPF and adult rat spinal cord tissue. (**a**) Mean stress-strain curves of the linear region from three different regions of the cord, namely lumbar, thoracic and cervical regions (N = 3 BRs, n = 3 TRs/BR). The lumbar region has the highest resistance to applied stress. (**b**) Mean curves of the linear region of stress-strain curves for: thoracic spinal cord tissue, DAPF/*B. mori* silk single fibre, Polyglactin filament and Axoguard^®^. The stress/strain curves of DAPF and *B. mori* silk filaments (n = 7 TRs) were closest to those of rat spinal cord tissue. (**c**) The accelerated *in vitro* degradation test showed a statistical significant decrease in maximum force of DAPF after 40 and 60 days (n = 7 TRs).

To compare the stiffness (the Young’s modulus) of DAPF and spinal cord, we first carried out uniaxial tensile tests to investigate the stress-strain correlation of adult rat spinal cord at lumbar, thoracic and cervical regions. We found that the spinal cord stiffness ranged from 145 to 210 kPa, with the lumbar level having higher stiffness than the other two regions (Fig. 5a and Supplementary Fig. S6). Then, we measured the stiffness of DAPF using the same strain rate as for the cord, finding 11 and 7.5 GPa in dry and wet states respectively (data not shown). Similarly, we measured the stiffness of *B. mori* filaments and of commercially available synthetic materials for peripheral nerve injury repair. Our findings demonstrated DAPF had a stress-strain behaviour significantly different from that of synthetic materials but more like that of spinal cord thoracic region (Fig. 5b and Supplementary Fig. S7).

### *A. pernyi* silk degrades gradually

Finally, a resorption study of DAPF was carried out *in vitro* to record any change of the mechanical strength of DAPF over a 60-day period (Fig. 5c). DAPF immersed in PBS at 70 °C showed gradual decreases in both the maximum force (Fig. 5c) and force-strain curves (Supplementary Fig. S8). *In vivo* degradation of DAPF was also assessed and we found an approximately 50% decrease in their weights after 5 months  implantation under the dura of the spinal cord of non-SCI adult rats (Supplementary Fig. S9).

## Discussion

Our findings demonstrated that DAPF promoted cell adhesion, contact guidance and rapid neurite extension in the two types of CNS neurons studied. This is the first time that the potential of DAPF to support CNS neuron growth has been demonstrated. The following features may account for these properties by increasing existing neurite length and promoting new filopodia formation in the growth cones^14^: (1) there is good evidence that nanotopography can influence cell axis alignment^15^; (2) the surface of DAPF showed a high density of longitudinally-orientated nanogrooves as shown by SEM (Supplementary Fig. S2); (3) a critical density of RGD tripeptide on surfaces optimises cell spreading, migration, and focal adhesion dynamics of rat fibroblasts^16^ and optimises surface expression of activation markers in bone marrow derived dendritic immune cells^17^; and (4) thoroughly degummed surfaces of DAPF are likely to have a high density of cell-adhesive RGD repeats. RGD is a cell recognition motif present in large quantities in ECM molecules such as fibronectin. It mediates cell attachment via different cell adhesion molecules, including integrins. CNS neurons have abundant integrins and can respond to RGD. Therefore, we propose that DAPF might prove useful for bridging the injury site while its natural decoration with RGD may promote injured spinal cord axons to adhere and regrow. Furthermore, our findings demonstrated that HUVECs also adhered to DAPF. These cells have abundant integrins and have been used as *in vitro* models for assessing angiogenesis^18^ and for testing the bioactivity of angiogenic factors in the context of spinal cord injury^19^. This highlights the need for future *in vivo* studies with DAPF scaffolds to assess blood vessel formation in parallel with axon regeneration.

Biocompatibility is a key aspect for any biomaterial designed for spinal cord repair. The disadvantages of other non-silk natural biomaterials include their potential immunogenicity and variability. Our *in vitro* and *in vivo* data demonstrated that DAPF induced no activation of^19^ microglia. This suggests that DAPF are unlikely to cause an inflammatory response and foreign body reaction when used in spinal cord repair. The excellent biocompatibility of DAPF observed here may result from the thorough degumming process effectively removing sericin, a common source for immune responses. Additionally, these data align with our previous work on peripheral nerve injury repair showing minimal activation of macrophages by DAPF^10^ and with other evidence showing thin films prepared from AP silk did not increase expression of inflammatory cytokines by macrophages and osteoblasts^20^. A recent study revealed an upregulation of proinflammatory cytokines by human monocyte-derived dendritic cells, which share a similar lineage to macrophages, when exposed to DAPF-knitted mats^21^. This suggests that material stiffness could influence the activation of immune cells, as the stiffness of DAPF-knitted mats would be significantly higher than that of single fibre or thin film of DAPF. Among other types of glial cells we have also conducted pilot experiments culturing primary rat astrocytes on DAPF. Preliminary observation of GFAP immunofluorescence and cell morphology revealed minimal signs of astrocyte activation when they adhered to DAPF (data not shown). In depth *in vitro* and *in vivo* studies of astrocytes interaction with DAPF will be carried out in future to confirm this.

There is growing awareness of the significance of biomechanical effects in tissue regeneration^22^. It is important that any biomaterial designed for spinal cord repair should be soft enough not to compress surrounding tissue and its mechanical stiffness should match or be appropriate to the spinal cord. Our data demonstrated that the stiffness of DAPF more closely matched that of adult rat spinal cord tissue compared with synthetic and natural materials used for peripheral nerve repair. Direct mechanical comparison with hydrogel materials was not conducted because of inherent differences in the nature of the materials and because of distinct differences in the types of mechanical tests they require (i.e. tensile tests for fibres and frequency sweep tests for hydrogels).

The stiffness match of DAPF with spinal cord tissue may be highly beneficial for regeneration, in fact *in vitro* and *in vivo* evidence indicates that mechanical mismatch at the interface between biomaterials and glial cells in the CNS triggers glial cell activation leading to an immune response and a foreign body reaction thought to contribute to glial scar formation^23^. In this respect, we have already noted our own evidence (Fig. 4) that DAPF did not activate microglial cells *in vitro* and *in vivo*. We further note that an optimum substrate stiffness *in vitro* beneficially influences neurite length and the gene expression in neural cells derived from a pluripotent cell line^24^. In this regard, we have shown that the mechanical stiffness of DAPF does not appear to adversely influence neurite outgrowth compared to standard *in vitro* growing conditions (Fig. S4). However, further evidence will be sought in future with *in vivo* SCI models and future designs of DAPF. In addition, we demonstrated that the tensile strength of DAPF was sufficient to prevent them breaking during or after implantation.

The mechanical properties of DAPF can be compared to electrospun nano-fibre biomaterials, which are more commonly researched. Synthetic electrospun fibres have good mechanical strength and degradation profiles but the solvents used in producing them are highly toxic and their fibre size is highly variable, with negative impacts on biocompatibility and neuronal outgrowth^25^. In contrast, electrospun nano-fibres produced from natural polymers degrade too quickly to provide sufficient mechanical strength to be useful in the context of spinal cord repair^26^.

Biodegradation is an important aspect to consider in biomaterial design for spinal cord repair. Biomaterial scaffolds should temporarily support regenerating axons and then degrade over time once that purpose has been met. A gradual degradation of the material prevents chronic immune responses and avoids removal surgery^27^. Moreover, there is evidence showing that axon infiltration into a scaffold can occur as early as one week post implantation into injured spinal cord and continue for several weeks^12^. Therefore, our data indicate that the degradation profile of DAPF may be suitable for spinal cord repair. Our results are in line with other evidence showing morphological and conformational changes in AP silk following enzymatic degradation by protease XIV^28,29^, as well as with our previous evidence showing a gradual degradation of DAPF implanted in injured peripheral nerves^10^.

The lack of therapies for spinal cord injury creates a substantial, unmet, clinical need for millions of patients. The evidence collected in this study suggests that DAPF possess a portfolio of material and biochemical properties that make them eminently suitable for use as a structural component for a spinal cord repair device in the future. Furthermore, these properties suggest that DAPF may conceivably also have potential for repairing brain damage, for example, by guiding axon growth in neurons derived from stem cells.

## Methods

All procedures involving the use of animal tissues were approved by the ethics committee of the University of Aberdeen and performed in accordance with the UK Home Office regulation - the Animals (Scientific Procedures) Act 1986.

### Preparation of DAPF and *B. mori* filaments

Demineralized, degummed and cleaned DAPF and *B. mori* filaments were used as supplied by Oxford Biomaterials Ltd who uses a proprietary process^30^. Briefly, the cocoons were soaked in aqueous 1 M ethylenediaminetetraacetic acid, pH adjusted to 10, for 72 h at 40 °C. Then the samples were washed 3 times for 3 h with running tap water. DAPF and *B. mori* filaments were reeled in the same way using previously reported standard methods in water and at room temperature (RT). Reeled silk baves were enzymatically degummed to remove sericin, washed, air dried and stored at RT until further use.

### Primary cortical neuronal culture

Cortices of Sprague Dawley rats at postnatal days 0–1 were harvested as a source for cortical neurons. The tissue was dissociated enzymatically with 50 U papain (Worthington) in Retinal Buffer solution at pH7.4 for 30 min at 37 °C. The enzymatic action was stopped with fetal bovine serum and cells were resuspended in Neurobasal medium supplemented with B-27 for up to 48 h (all from Gibco Life Technologies). To study the interaction between neurons and DAPF, custom-made tissue culture dishes were designed (Supplementary Fig. S1). Three individual silk filaments were placed in parallel on 22 mm round glass coverslips with their ends folded over coverslip edges and glued at the back with Super Glue Gel (RS Components). Holes with a diameter of 13 mm were punched in the bases of tissue culture dishes and the coverslip, silk side-up, was glued with RTV 3140 (Dow Corning) to the underside of the dish, sealing the punched hole. To allow consistent counting, approximately 12.5 × 10^5^ of neurons were seeded on the 13 mm diameter area containing the filaments, and cultured for 2 to 5 days, followed by fixing with 4% buffered paraformaldehyde solution (PFA) and processing for immunocytochemistry. For RGD adhesion assays, cortical neurons were plated in the same way but cultured for only 1 h with and without RDG tripeptide (50 µM; Sigma) in the culture media.

### Host immune response to DAPF

For *in vitro* studies, primary microglia were cultured as described above^31^ from the cortices of Sprague Dawley rats at postnatal days 3–6 and plated on tissue culture dishes with DAPF. Approximately 2 × 10^4^ cells were seeded in each dish. After 48 h in culture, cells were fixed with 4% PFA and processed for immunocytochemistry. Cultures without DAPF received lipopolysaccharide (LPS, 1 µg/mL; Sigma) to serve as positive controls (indicated as “LPS” in Fig. 4). Cultures without DAPF and without treatment of LPS served as negative control (indicated as “Control” in Fig. 4). All culture media were collected and stored at −20 °C in sealed containers for later examination. The amount of NO released by microglia in culture media was quantified by measuring the level of nitrite (-NO_2_), a stable metabolite of NO. Nitrite release was measured in triplicate with the Griess assay, which determines the nitrite content using an NaNO_2_ standard curve. For *in vivo* studies we have provided a detailed description of the surgery in the Supplementary methods. Briefly, at 5 months, rats with DAPF implantation and naïve control rats (n = 3) were perfused with 4% PFA and the spinal cord directly underneath the DAPF was harvested and processed for immunohistochemistry.

### Tissue processing and neurochemical staining

Cell cultures were fixed with 4% PFA in phosphate buffered saline (PBS) for 20 min, followed by incubation with 10% goat serum for 1 h at all at 20 °C. Cells were then incubated overnight at 4 °C with appropriate primary antibodies including mouse anti β-Tubulin III (1:1000; Sigma) for cortical neurons, rabbit anti Iba-1 (1:1000; Wako) for microglia, and mouse anti-iNOS/NOS Type II (1:200; BD Biosciences) for activated microglia. Following 3 washes with PBS, cells were incubated at 20 °C for 2 h at room temperature with appropriate secondary antibodies including goat anti-mouse/rabbit Alexa Fluor 488 and goat anti-mouse Alexa Flour 555 IgG2a (all at 1:400; Invitrogen). Cell nuclei were stained with Hoechst 33342 (2 µg/ml in PBS; Sigma). For *in vivo* immune response study the harvested spinal cords were post-fixed with 4% PFA overnight at 4 °C. The tissue was then cryoprotected with 30% sucrose in PBS and embedded in OCT medium. Transverse sections at 14 µm thickness were prepared using a cryostat (Leica). Sections were then incubated with 10% goat serum followed by incubation with the Iba-1 primary antibody overnight at 4 °C. Following 3 washes with PBS, sections were incubated with goat anti-rabbit Alexa Fluor 488 for 2 h at 20 °C. Sections were mounted with PBS/glycerol (1:8 ratio) after counterstaining with Hoechst. All primary/secondary antibodies and goat serum were prepared with PBS containing 0.2% Trition X-100 (Sigma) and 0.1% sodium azide (Sigma).

### Scanning electron microscopy

Silk and non-silk based materials were aligned parallel to each other on a stub to observe the outer surface and embedded vertically in a Leit-C Plast conductive adhesive paste (Agar Scientific) to observe and measure cross-sections. For cortical neuron morphology on DAPF, cells were cultured, as described above, for 5 days and then fixed with 2.5% glutaraldehyde buffered with cacodylate, post-fixed with buffered 1% osmium tetroxide solution, dehydrated in an ethanol series and critical-point dried using hexamethyldisilazane. All samples were sputter coated with gold/palladium using Q150T Turbo-Pumped Sputter Coater (Quorum Technologies) and imaged with an EVO MA10 scanning electron microscope (SEM; Carl Zeiss AG).

### Imaging and image analysis

All fluorescence images were captured with an epifluorescence microscope (Nikon) or a confocal microscope (Zeiss). Live cell imaging was performed using the Nikon Eclipse Ti-E microscope equipped with a stage incubator chamber (Oko-Lab) to ensure optimal culture conditions (37 °C, 90% humidity and 5% CO_2_). Time-lapse images were collected with a Digital Sight DS-U3 controller using NIS-Elements Advanced Research software version 4.3 (Nikon). To assess cortical neuron outgrowth, the longest neurite length per neuron was measured using either Neuron J (ImageJ plug-in, NIH) for cells cultured on DAPF or HCA Vision (CSIRO Biotech Imaging Group) for cells cultured on glass coverslips. For cell adhesion studies, the total number of cells attached on each DAPF or *B. mori* silk filament was counted manually and blindly. To analyze iNOS immunoreactivity, images from 5 areas along each fibre were randomly selected and taken at 20x Objective magnification, and the numbers of cells that were immunoreactive for both Iba-1 and iNOS were counted using Photoshop (Adobe). We classified Iba1 immunoreactive microglia in the dorsal horn as having “resting” morphology when their process lengths were double the soma diameter. In contrast, we classified Iba1 immunoreactive microglia in the dorsal horn as having “effector” (activated) morphology when their process lengths were less than double the soma diameter^2^.

### Tensile test procedures

To determine the tensile properties of rat spinal cords, we harvested fresh spinal cords (n = 3) from male Sprague Dawley rats (8 weeks of age) for comparison with silk filaments and synthetic polymers used for peripheral nerve repair. Each cord was separated into cervical (~C1 to C7), thoracic (~T1 to T12) and lumbar (~L1 to L6) segments. The tissue was suspended with custom-made clamps to prevent slippage and tested within 2 h from harvesting. The crosshead was lowered until the load cell just started to respond and the diameter of the segment was measured with digital calipers to estimate the cross-sectional area. The tensile properties of DAPF and *B. mori* filaments were tested in bundles (~7 filaments) or single filaments. They were mounted on custom-made cardboard frames giving an initial grip to grip separation of 5 mm. For comparison of tensile properties of DAPF the analysis was restricted to fibre-like biomaterials only. Synthetic and natural materials used here were: 1) Polyglactin 910 resorbable monofilament sutures (Ethicon) and 2) AxoGuard® nerve connector produced from porcine submucosa extracellular matrix (AxoGen). These are non-silk based materials commonly used in the treatment of peripheral nerve injury.

For tensile testing we used a Z0.5 tensile tester (Zwick Roell) with 5 N or 40 N load cells. Strain rate for all specimens was set to 20% min^−1^ until failure. To make the results comparable, different spinal cord regions were tested at the same strain rate as that used for the biomaterials. All data process was performed using MATLAB 8.3 (MathWorks). Young’s modulus was calculated using the method detailed in Supplementary Materials. Ultimate Tensile Strength was derived from stress-strain curves and taken as the highest stress before failure. Maximum force was measured from force-strain curves and taken as the greatest force before failure. Representative nominal stress-strain curves were plotted for all tests. Force-strain curves were plotted instead where test samples lacked a uniform cross-sectional.

### Degradation testing for DAPF

Degradation of DAPF was carried out both *in vitro* and *in vivo*. An accelerated *in vitro* degradation test was conducted according to the ISO standard for polymeric medical devices (ISO 10993-13:1998). Briefly, DAPF were incubated at 70 °C in PBS in a sealed container in a thermostatic oven (Binder) for 1, 20, 40 and 60 days. The tensile properties were tested within 2 h from the end of the incubation period. The method for *in vivo* degradation is described in the Supplementary Materials.

### Statistical analysis

Data were presented as mean ± standard error of the mean (SEM). Statistical analyses were performed using Prism 5 (GraphPad Software Inc.). Following a test for normal distribution (D’Agostino and Pearson omnibus), data were analyzed using a one-way ANOVA with post hoc Bonferroni t test unless otherwise stated in the figure legend. Statistically significant differences were considered at *p < 0.05, **p < 0.01, and ***p < 0.001.

### Data availability statement

All data generated or analyzed during this study are included in this published article (and its Supplementary Information files).

## Electronic supplementary material

Supplementary Materials
