# Supplementary material for: The potential of Antheraea pernyi silk for spinal cord repair
Source: Sci Rep. 2017 Oct 23;7:13790. doi: 10.1038/s41598-017-14280-5 (PMC5653809; doi:10.1038/s41598-017-14280-5)
Supplement: Supplementary file 1 — Supplementary Materials [file 41598_2017_14280_MOESM1_ESM.pdf]

Supplementary Materials

The potential of *Antheraea pernyi* silk for spinal cord repair

A. Varone[1], D. Knight[2], S. Lesage[2], F. Vollrath[2,3], A.M. Rajnicek[1], W. Huang[1,*]

[1]Institute of Medical Sciences, University of Aberdeen, Aberdeen, AB25 2ZD, UK

[2]Oxford Biomaterials Ltd., Magdalen Centre, Oxford Science Park, Oxford, OX4 4GA, UK

[3]Department of Zoology, University of Oxford, Oxford, OX1 3PS, UK

*Corresponding author; E-mail: w.huang@abdn.ac.uk

1. Supplementary methods

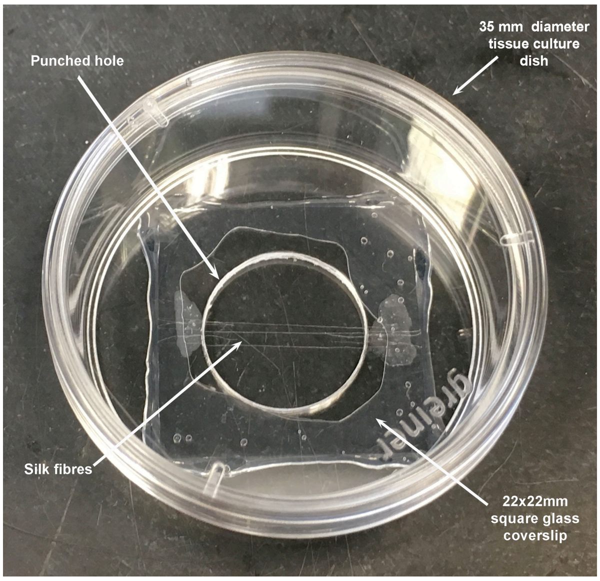

Figure S1. Example of DAPF culture dish preparation. Photo of a customised tissue culture dish for testing cell growth on DAPF. The ends of three individual DAPF were glued in parallel on a glass coverslip and the coverslip was glued, DAPF side facing the dish to cover a hole punched previously in the base of the dish. This makes a shallow well in the base of the dish with the fibres exposed to the culture medium, ready for medium addition and cell seeding.

Primary *Xenopus* neuron cultures

Xenopus laevis spinal cord neurons were cultured as described previously (Rajnicek et al., J Cell Sci 2006; 119:1723-35). The neural tube was isolated from stage 20-22 embryos, dissociated by trituration sterile Pasteur pipettes and cultured in 77% Steinberg's solution, 20% Modified L-15, 2% PenStrep and 1% FBS with 1% calf serum for up to 10 h.

HUVEC cultures

To investigate vascularization, human primary umbilical vein endothelial cells (HUVEC), a gift of Dr Neil Vargesson (the University of Aberdeen), were grown to confluency in DMEM high glucose with 10% FBS and were discarded after passage 9. HUVECs were fixed with 4% paraformaldehyde in PBS for 20 min at room temperature and processed for immunocytochemistry. A fluorescent dye Rhodamine Phalloidin (1:200; Molecular Probes) was used to label F-actin in HUVECs.

Degradation of DAPF *in vivo*

This formed part of the study that examined the host immune response to DAPF as described in the methods of the main text of the manuscript. Twelve adult male Wistar rats were used (2 naïve, 5 for three months and 5 for five months DAPF implantation). Briefly, a partial laminectomy was performed under general anesthesia (isofluorane: 3% induction and 1-2% maintenance) by removing the spinous process of T10 and then the left side of the lamina of T10 using a fine tip rongeur. A small slit was carefully made in the exposed dura using fine spring scissors, without touching the cord. A weighed sample of DAPF was inserted directly underneath the dura and on top of the T10 cord. The dura was sutured after implantation using 7-0 sutures (Vicryl, Ethicon, UK). SCI was not performed on any of these animals. After 3 and 5 months respectively, naïve animals and animals with implanted DAPF were perfused with 4% PFA and the DAPF implant was removed carefully, and inspected for signs of degradation by assessing its weight loss and by bright-field microscopy.

Script designed for Young's modulus calculations

```
strain= a(:,1)/100;

force=a(:,2);

area= (x)m^2;

pressure=force/area;

pressure=pressure/1e6;

for j=1:length(strain)

for i=1:j

    if(strain(i)>0.02 & strain(i)<0.03)

        x(i)=strain(i);

        y(i)=pressure(i);

    end

end

end

plot(strain,pressure)

xlabel ('strain [-]','FontSize',20)

ylabel ('stress [MPa]','FontSize',20)

title ('2-SP-filamentsbundle-WET','FontSize',20)

set(gca,'XTick',[0:0.03:1])

set(gca,'XTickLabel',[0:0.03:1]);

set(gca,'FontSize',16);

x(x==0) = [];

y(y==0) = [];

p=polyfit (x,y,1);

slope=p(1)

figure

plot(x,y)
```

The above script was developed and executed using Matlab.

2. Supplementary results

2.1 Representative SEM images of DAPF.

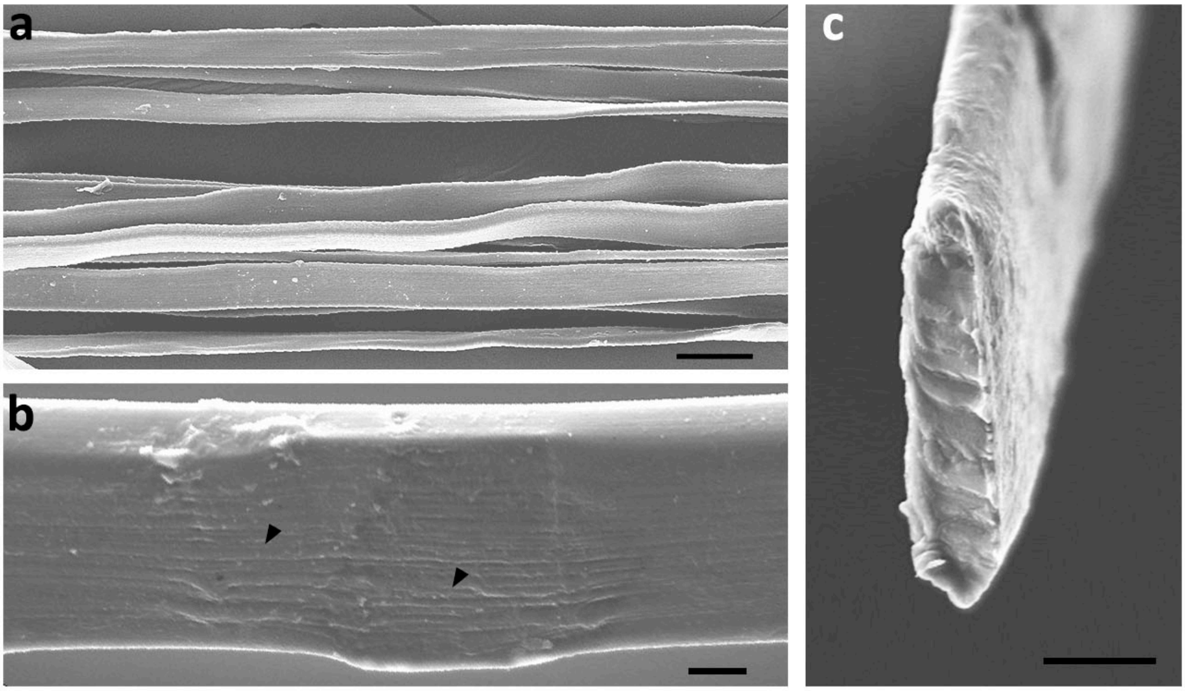

Figure S2. High magnification views of DAPF with SEM. *(a)* Longitudinal view of a bundle of DAPF. *(b)* The 'flat' surface showing parallel microgrooves (black arrowheads). *(c)* A cross-section view of a DAPF single fibre. Scale bars = 50 µm *(a)* and 10 µm *(b & c)*.

2.2 Detailed analysis of CNS neuron-DAPF interactions

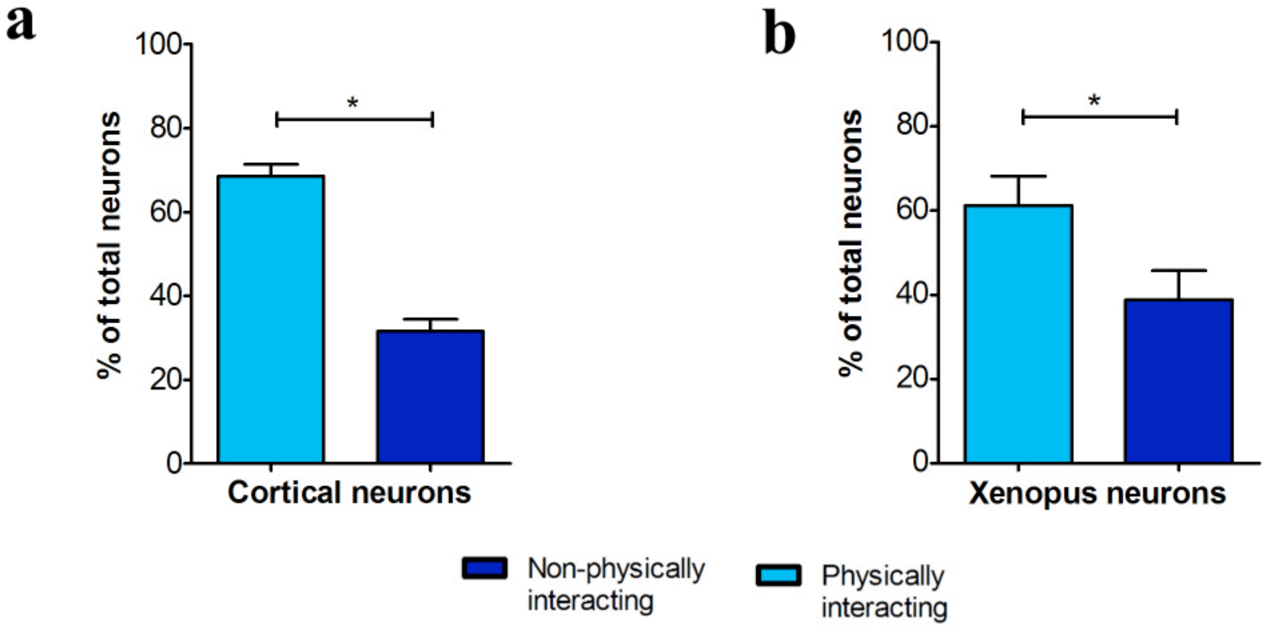

Figure S3. Interactions of CNS neurons with DAPF. We described cortical neurons and *Xenopus* neurons within 100-µm distance from both sides of each filament as either physically interacting, i.e. cell bodies in direct contact with the filament, or non-physically interacting, i.e. no direct contact of cell bodies with the filament (N = 7, n = 5). Our analysis showed that the numbers of CNS neurons physically interacting with DAPF were significantly higher than those of CNS neurons non-physically interacting with DAPF (a & b; *p<0.05, *t-test*). N = biological replicates (BRs), n = technical replicates (TRs) per BR.

2.3 Neurite length analysis of cortical neurons grown on DAPF and on PDL substrate

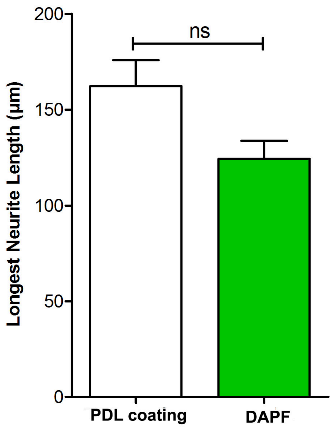

Figure S4. Quantitative analysis of the longest neurite length under DAPF and PDL conditions. We compared the mean longest neurite lengths of cortical neurons grown on glass coverslips coated with PDL and on DAPF without any coating. There was no significant (ns) difference in the mean longest neurite length per neuron between the two conditions (N = 3 BRs, n = 3 TRs; $p>0.05$, *t-test*). PDL = poly d-lysine.

2.4 Cortical neuron adhesion on DAPF and *B. mori* silk filaments

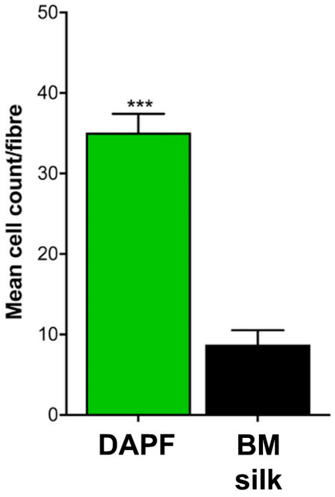

Figure S5. Mean cell count per filament of cortical neurons adhering to DAPF and *B. mori* (BM) silk filaments. We observed that the number of cells attached to DAPF was significantly higher than that attached to *B. mori* silk filaments (N = 3 BRs, n = 3 TRs; ***$p<0.001$ vs BM silk, *t-test*).

2.5 Mechanical properties of the adult rat spinal cord

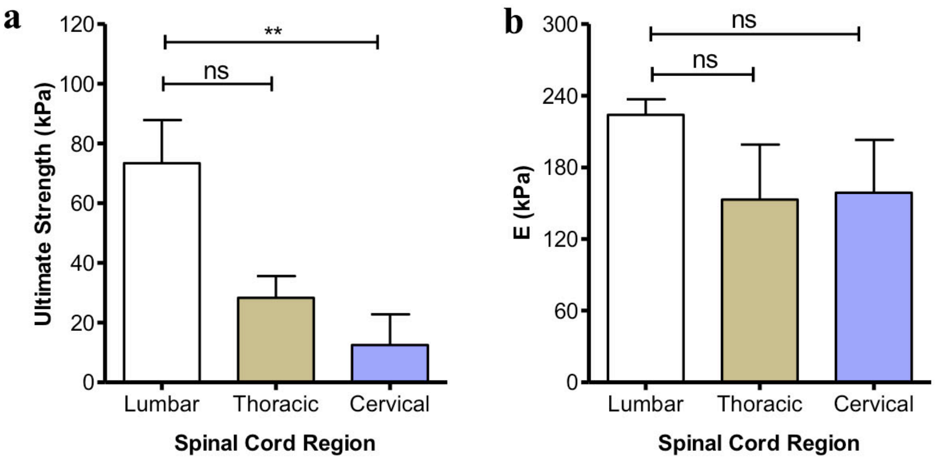

Figure S6. Tensile behaviour of adult rat spinal cord tissue. a. The ultimate strength for the lumbar spinal cord was significantly higher than that for the cervical spinal cord (N=3 BRs, n = 5 TRs, **$p<0.01$). **b.** Lumbar, thoracic and cervical spinal cords showed no significant differences in Young's modulus (E) for the linear part of the stress-strain curves (N=3 BRs, n = 5 TRs; $p>0.05$).

2.6 Mechanical properties of silk and non-silk biomaterials

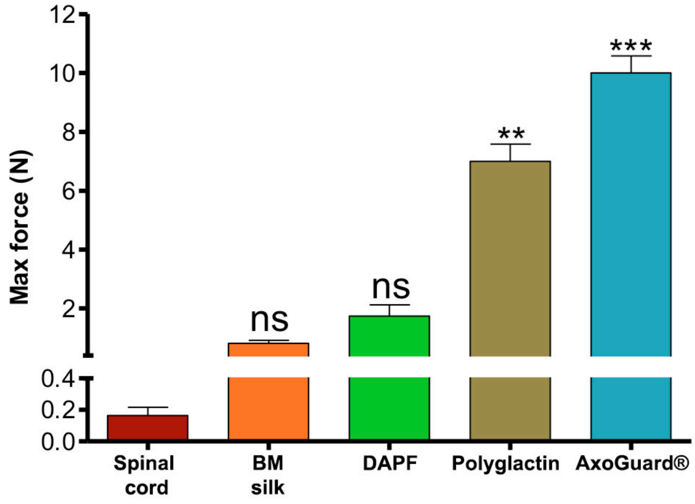

Figure S7. Comparison of max force values among: spinal cord, silk, and non-silk biomaterials. The max forces of DAPF and BM silk filaments were not significantly different than that of adult rat spinal cord tissue ($p>0.05$). In comparison, the max forces of the two non-silk

biomaterials examined here were significantly higher than that of the silk and spinal cord tissue (n=7 TRs, **p<0.01 and ***p<0.001 *vs* silk and spinal cord).

2.7 Degradation of DAPF *in vitro*

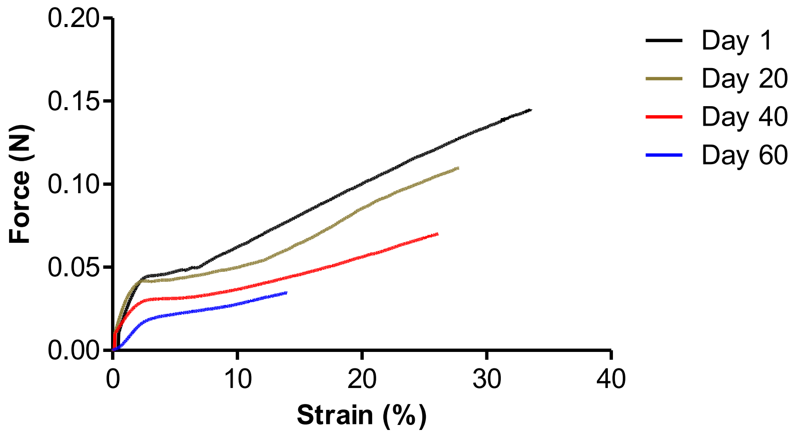

Figure S8. Temporal degradation profile of DAPF *in vitro*. Analysis of the force-strain responses following tensile tests of individual filament revealed a decrease in the strength of DAPF, which was proportional to the time of degradation (n = 7 TRs).

2.8 Degradation of DAPF *in vivo*

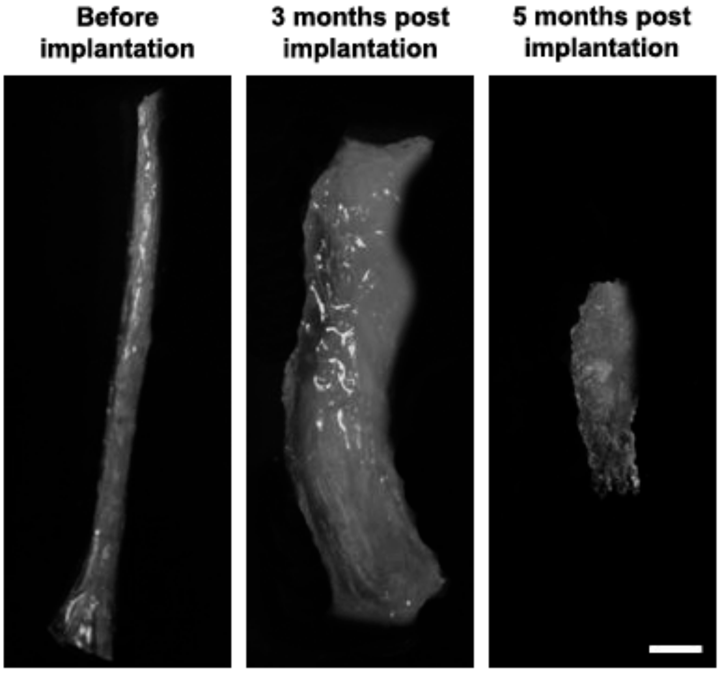

Figure S9. Photos of DAPF following *in vivo* implantation. Bright-field microscopic images show a bundle of DAPF that has not been implanted *(left)*, that has been implanted for 3 months *(middle)* and for 5 months *(right)*. Significant signs of degradation were noticed after 5 months implantation. Scale bar = 750 μm.
